# Relationship Between Orthodontic Treatment Need and Oral Health-Related Quality of Life among 11–18-Year-Old Adolescents in Lithuania

**DOI:** 10.3390/ijerph15051012

**Published:** 2018-05-17

**Authors:** Aistė Kavaliauskienė, Antanas Šidlauskas, Apolinaras Zaborskis

**Affiliations:** 1 Department of Orthodontics, Faculty of Odontology, Medical Academy, Lithuanian University of Health Sciences, A.Lukšos-Daumanto Street 6, LT-50106 Kaunas, Lithuania; antanas.sidlauskas@lsmuni.lt; 2 Department of Preventive Medicine & Health Research Institute, Faculty of Public Health, Medical Academy, Lithuanian University of Health Sciences, Tilžės Street 18, LT-47181 Kaunas, Lithuania; apolinaras.zaborskis@lsmuni.lt

**Keywords:** oral health-related quality of life, child perception questionnaire, malocclusion, orthodontic treatment need, associations, adolescents, Lithuania

## Abstract

The aim was to examine the relationship between orthodontic treatment need and Oral Health-Related Quality of Life (OHRQoL) among Lithuanian adolescents aged 11–18 across gender and age groups. A representative cross-sectional study of 911 adolescents aged 11–18 (mean (M) = 15.53; Standard Deviation (SD) = 1.52) was conducted in 26 public schools. The schoolchildren completed the Child Perceptions Questionnaire to evaluate their OHRQoL. The Index of Orthodontic Treatment Need (IOTN) was used to evaluate the severity of malocclusion. The strength of association between variables was evaluated via negative binomial regression estimating the ratio of sum score means (RSSM). A worse OHRQoL score was associated with a higher grade of IOTN; however, only the Emotional and Social wellbeing domains were significantly affected by malocclusion (RSSM = 1.158; 95% Confidence Interval (CI): 1.083–1.237 and RSSM = 1.205; 95% CI: 1.114–1.304, respectively). The significant association was identified only among females (RSSM = 1.264; 95% CI: 1.176–1.359). A significant association was observed in all age groups for Emotional and Social well-being domains but only in the oldest age group for Oral Symptoms and Functional Limitations domains. Conclusions: Malocclusion has a negative impact on the OHRQoL of young people with emotional and social aspects being the most affected. Girls and older adolescents suffered from malocclusion more than boys and their younger counterparts.

## 1. Introduction

Over the last decades, the interest in quality of life (QoL) as a measurement tool has grown considerably in the field of medicine, including dentistry, and is referred to as ‘Health-Related Quality of Life’ According to the World Health Organization (WHO), the term describes an individual’s assessment of how the following factors affect his or her well-being: experience of pain/discomfort, physical function, psychology, and social function [1]. This definition was later adapted to dentistry as 'Oral Health Related Quality of Life' (OHRQoL) [2,3,4]. Studies in adult populations generally report an association between malocclusion/need for orthodontic treatment and OHRQoL scores [5,6]. Quantifying the negative impact caused by malocclusion helps clinical orthodontists be more attuned to the needs of their patients and allows them to provide quality treatment which meets the patients’ expectations. At the population level, the identification of weak points within OHRQoL is important as it helps to uncover the needs of individuals in the society and thus direct public health actions and policies more effectively toward the prevention and treatment of occlusal disorders [4,7,8].

When studying OHRQoL in adolescence, the negative impacts associated with malocclusion can be particularly relevant. Adolescence is a critical time of identity formation marked by physical, psychological and social changes, so all peculiarities inherent to this phase of life should be taken into consideration [9]. The WHO defines adolescents as those between 10 and 19 years of age [10]. Adolescence may be roughly divided into early (10–13 years), middle (ages 14–16), and late (17–19 years) adolescence stages [11], with the knowledge that adolescents of the third stage are very different from those of the first stage [12].

The factors that influence OHRQoL in adolescents have been examined in the last decade [13,14,15,16,17]. There are several studies based on the relationship between malocclusion and OHRQoL using representative population-based samples that control for a set of confounding factors when reporting the association between predictors and the outcome [13,17,18,19]. The most frequently investigated confounding factors were socio-demographic factors such as gender, household income and parents’ education. Because socio-demographic factors may influence OHRQoL, the nature and magnitude of impacts may in fact vary between populations of different cultural backgrounds [20,21]. Therefore, similar studies conducted in varying countries, including Lithuania, have their own inherent value and may be of interest. 

In the research of the impact of malocclusion on OHRQoL, it is important to gather accurate and reliable data about the severity of orthodontic anomalies. Therefore, the evaluation of changes in normal occlusion by indices seems to be necessary. Several indices have been introduced and used to rank or categorize the severity and complexity and to evaluate the treatment need and outcome of malocclusion [22,23,24,25,26,27]. Among indices employed for this purpose, the Index of Orthodontic Treatment Need (IOTN), which was formulated in the U.K. by Shaw (1989), has gained international acceptance [28]. The IOTN was most frequently employed in classifying the study population clinically [6]. It is valid, reliable and easy to use to rank malocclusion in terms of the significance of various occlusal traits for an individual’s dental health and perceived aesthetic impairment as well as aiming at identifying those individuals who will most likely benefit from an orthodontic treatment [22,28,29]. The index has two components: aesthetic (AC) and dental health (DHC). The AC of IOTN is a self-perceived index of dental attractiveness which can help to evaluate changes of attractiveness by malocclusions, while DHC is based on the severity of individual occlusal traits. The DHC allows the patients to be classified into one of five groups (grades): no need for treatment, slight need for treatment, moderate/borderline need for treatment, need for treatment, and definite need for orthodontic treatment. The IOTN-DHC has been used in several studies exploring an association between OHRQoL and severity of malocclusion [17,30,31,32,33,34]. 

Next, QoL is a ‘dynamic construct’, and the value attributed to any domain of OHRQoL may change over adolescence [35,9]. Therefore, age should be considered a predictor of OHRQoL, and the cutoff points of age subgroups should be investigated [13]. Most studies which explored the association between adolescent malocclusion and OHRQoL were conducted in samples of adolescents aged 11–14 years, e.g., the Child Perception Questionnaire (CPQ_11–14_), proposed by Jokovic et al. as a tool to measure OHRQoL, was specifically designed for adolescents of this age [36]. The authors of the instrument discussed the role of children and adolescents’ cognitive abilities in self-reported health status and suggested age-specific questionnaires to be used only for children younger than 11 years old. Only a few relevant studies were conducted among adolescents over the age of 14 but none in populations older than 16 years of age [37,38,39,40]. Therefore, we hypothesized that the association between malocclusion and OHRQoL in older adolescent samples (e.g., in 15–16- or 17–18-year-olds) is possibly more evident than in samples of adolescents aged 11–14 years. 

Gender differences in QoL have been well established, with higher life satisfaction scores among boys than among girls across all age groups in adolescence [41]. Several studies have also demonstrated that boys had more positive experiences of OHRQoL than girls, especially in emotional and social wellbeing domains [13,14]. The association between malocclusion and OHRQoL may also be influenced by gender; however, literature on this issue is scarce.

Finally, the OHRQoL of adolescents demands further research for several reasons. First, although several hypotheses on the association between malocclusion and OHRQoL have been proposed and tested, a consensus has not yet been reached [13]. One of the reasons for explaining this failure might hinge upon the choice of an inappropriate model (analysis method) to examine the association, since this association is much more complicated than a simple linear one. 

Further studies are therefore needed to provide additional evidence concerning these matters. As such, the aim of this study was to examine the association between orthodontic treatment need and OHRQoL among Lithuanian adolescents aged 11–18 years and to identify potential differences across gender and age groups. 

## 2. Materials and Methods 

### 2.1. Ethical Statement

The study conformed to the principles outlined in the Declaration of Helsinki. Ethical approval for the study was granted by the Kaunas Regional Biomedical Research Ethics Committee (reference number BE-2-27) and was in line with local practice for school survey distribution. Written informed consent for a child’s participation in the study was sought from both parents prior to his/her participation in the research.

### 2.2. Sample, Participants and Data Collection 

This study was an observational study with cross-sectional design and targeted adolescents aged 11 to 18 years. A minimal number of 260 participants was calculated to be sufficient for testing statistical hypotheses about relationships between orthodontic treatment need and CPQ sum scores in each age group of adolescents in the present analyses (in three age groups, *N* = 3 × 260 = 780). This calculation was produced with the software G*Power 3.1 for Poisson regression with z test (one tail) and other input parameters: α = 0.05; 1 − β = 0.8; Exp(β/H_1_) = 1.1 (‘expected effect’); Exp(β/H_0_) = 1 (‘no effect’); Mean exposure (λt) = 10; Binomial *X* distribution with π = 0.5 [42]. However, the data collection followed a larger sample size (*N* = 1454) that was estimated for the entire research project in Lithuania with more research objectives. 

The sample being studied was made up of students from 26 randomly selected public schools using random cluster (school, class) sampling. School authorities were contacted by researchers and informed about all aspects of the study. Parents were then asked to provide permission for their child to participate in the study. 

Data were collected using both questionnaires and dental examinations. The self-completed questionnaires were administrated in school classrooms before dental examination by the classroom teaching staff to ensure a familiar and consistent environment. Confidentiality and anonymity of respondents were ensured. The dental examinations were performed according to the methodology of oral status evaluation recommended by the WHO [43] under standardized conditions in the school’s medical offices using portable equipment for dental examination. The orthodontic examinations were a part of the dental examinations. All examinations were undertaken by one orthodontist (A.K.) who was trained and tested in reliability of accessing IOTN (U.K. Cardiff University School of Dentistry, 2012) and her assistant; therefore, inter-examiner reliability analysis was not needed in this study.

Before the main study, a pilot test was carried out with a sample (*N* = 48) of students in one school. It confirmed the feasibility of the methodology with only minor modification of questionnaire wording and confirmed the organization of the data collection procedures. 

In the main study, 911 students participated in both the questionnaire and dental surveys and provided necessary data for the present study. The response rate for the questionnaire survey was 80% and for the dental examinations, 68%. Maintaining the same fieldwork methods and conditions, the data were collected in the 2013/2014 school-year (*N* = 831) and 2016/2017 school-year (*N* = 80). 

### 2.3. Evaluation of Oral Health-Related Quality of Life

The CPQ was used for the evaluation of OHRQoL [37]. The Lithuanian version of the CPQ was prepared using the necessary translation pipeline [44]. A validation study demonstrated that the instrument conformed to the concepts of the original CPQ_11–14_ and had excellent reliability (Cronbach’s alpha was 0.89) [45]. The measures within the instrument had a significant association with the global life satisfaction among Lithuanian adolescents in all age groups [46]. The CPQ is a 37-item questionnaire consisting of four health domains (subscales), namely, oral symptoms (OS, 6 items), functional limitations (FL, 9 items), emotional well-being (EWB, 9 items), and social well-being (SWB, 13 items). The items are scored on a 5-point Likert scale ranging from 0 (“never”) to 4 (“every day or almost every day”). For each subject, sum scores were calculated by summing up responses to items in each domain, and CPQ sum scores were calculated by aggregating scores in all domains. Sum scores of OS ranged from 0 to 24, sum scores of FL and EWB ranged from 0 to 36, and sum scores of SWB ranged from 0 to 52. As such, in total, CPQ scores can range from 0 to 128. Higher sum scores referred to worse OHRQoL. In analyses, the sum scores of total CPQ and its domains were considered as outcome (dependent) variables. 

### 2.4. Evaluation of Orthodontic Treatment Need

In order to evaluate malocclusion, IOTN was recorded according to the methodology by Richmond (2008) [47]. In the present study, we used the DHC of IOTN. The measure categorizes the severity of malocclusion based on the relative effect of the various deviant occlusal traits on the longevity of the dentition. For the DHC of IOTN, 10 traits of malocclusion were assessed: overjet, reverse overjet, overbite, open bite, cross bite, crowding, impeded eruption, defects of cleft lip and palate as well as any craniofacial anomaly, Class II and Class III buccal occlusion, and hypodontia. Only the highest scoring trait was used to assess treatment need. The five grades were outlined. Grade 1 recorded small deviations from normal and was categorized as ‘no need of orthodontic treatment’. The deviant occlusal anomalies become more severe in Grades 2, 3 and 4, while grade 5 represented the most severe malocclusion (e.g., impacted teeth, large overjet greater than 9 mm, defects of cleft lip and palate) and was categorized as ‘very great need of orthodontic treatment’. Grades 4 and 5 were regarded as clinical need for treatment. In the analyses, the IOTN was considered as an independent variable of interest.

### 2.5. Previous or Present Orthodontic Treatment

In the questionnaire survey, respondents were asked whether they had ever noticed that their teeth were irregularly grew/situated, or they had malocclusion. Those respondents, who reported a possible or confirmed by dentist malocclusion problem, were asked additionally whether they have had orthodontic treatment. The question was asked separately in regard to dental plate and fixed orthodontic appliance (braces) therapy. The responses were: 1 = presently; 2 = previously; 3 = never. In the analyses, a group of adolescents who had current or previous orthodontic treatment by dental plate or fixed orthodontic appliance was selected. The remaining adolescents were admitted to the ‘untreated’ group, regardless of the need for orthodontic treatment. Participants with previous orthodontic treatment were not excluded from analysis. Due to this reason, data were adjusted for orthodontic treatment as a potent confounder in the association between IOTN and OHRQoL.

### 2.6. Socio-Demographic Variables

Some socio-demographic variables were chosen to be included in the present study due to their potential to confound the association between malocclusion and OHRQoL. They were gender (male and female), age groups (11–14 years old, 15–16 years old and 17–18 years old), and socioeconomic criteria defined by family affluence. 

Family affluence was measured by the Family Affluence Scale (FAS), which has been specially developed for studies among children and adolescents as a measure of social position of young people [48]. The scale is simple and easy to answer, even for young adolescents. The present FAS included four questions, which covered car and home computer ownership, own bedroom, and travelling on holidays. A composite FAS score was calculated for each respondent based on their responses to these four items, and then a three-point ordinal variable was composed for the present analysis, in which a score 0–3 indicated low affluence; score 4–5 indicated medium affluence, and score 6–7 indicated high affluence. 

### 2.7. Statistical Analysis

The distribution of sum scores was skewed to the direction of low values; consequently, it deviated substantially from the normal distribution but generally followed a Poisson distribution. Therefore, medians and interquartile ranges (IQR) were calculated. The null hypothesis that medians were the same across groups was tested using a median test. All reported *p* values were from two-sided statistical tests and *p* values ≤ 0.05 were considered statistically significant. 

The association between malocclusion and OHRQoL was explored using the Poisson model [49,50,51]

*λ* = exp(*B*_0_ + *B*_1_*X*_1_ + *B*_2_*X*_2_ + *B*_3_*X*_3_ + ...)

where *λ* is the mean value of outcome (dependent) variable and *X*_1_, *X*_2_, *X*_3_, ... are values of predictors (independent variables). *B*_0_ is an intercept and *B*_1_, *B*_2_, *B*_3_, ... are coefficients for corresponding predictors. A value exp(*B_i_*) = $e^{B_{i}}$ is a measure of the association strength, which indicates how many times the mean value of an outcome variable increases when the value of the *X_i_* predictor increases by one unit (it is an analogue of relative risk for binary outcomes). In the present analyses, the CPQ overall or domain sum score was chosen as the dependent variable, and the IOTN was chosen as an independent variable. These two variables consisted of a bivariate model. Furthermore, gender, age, FAS and orthodontic treatment were included in the model as adjusted independent variables in a multivariate model. The value exp(*B_i_*) then becomes the ratio of the CPQ sum score means (RSSM). The coefficients *B* were estimated using Negative Binomial Regression (NBR) analysis, which was chosen as a modified Poisson regression analysis to stem the problem of overdispersion (the variance of outcome variable exceeding the mean) [50,51]. The model fit to the existing data was assessed by deviance/df and Pearson *χ*^2^/df; values of these estimates close to 1 indicated a good model fit. In addition, the likelihood ratio *χ*^2^ in omnibus test with *p* < 0.05 shows that several independent variables were significantly related to the dependent variable [49,51]. The model was fitted and run separately for each domain and the overall CPQ using both sets of predictors, which resulted in 10 regression models. In addition, the model was tested in groups of adolescents by gender and age, adjusting data by FAS variable only. Furthermore, in order to ensure equal conditions of hypothesis testing in groups, the number of subjects was balanced using a data weighting procedure.

All analyses were performed using the SPSS statistical package (version 21; IBM SPSS Inc., Chicago, IL, USA, 2012). 

## 3. Results

The study included 911 participants between the ages of 11–18 (mean (M) = 15.53; Standard Deviation (SD) = 1.52), 40.6% male and 59.4% female. Data on family affluence were missing for only 2.5% of subjects who were then excluded from the multivariate analysis. Almost half of the adolescents in the sample were regarded as living in highly wealthy families. Detailed socio-demographic distribution of the study participants is presented in Table 1.

Table 1 also shows the distribution of study participants according to current or previous orthodontic treatment. With regard to dental plates, a total of 235 (36%) of participants reported using this therapy presently or have used it once before, commonly for 1 to 3 years. Usage of fixed orthodontic appliances was less prevalent; it was reported by 59 (6.5%) participants. Both methods of the therapy were combined into one characteristic ‘orthodontic treatment’ that indicated the present or previous orthodontic treatment regardless of the method. The proportions of ‘treated’ and ‘untreated’ participants were 29.3% and 70.7%, respectively. 

Table 2 shows distribution of IOTN points. Based on the criteria for assessment of the IOTN, the need for orthodontic treatment was 33.4% (95% CI: 30.3–36.5) of the study sample. There were no significant differences between males and females in the prevalence of IOTN points or in the need for orthodontic treatment (34.3% in males and 32.7% in females; *p* = 0.613). The frequencies of the IOTN points had a significant relationship with age and there was a small increasing trend of the need for treatment by age (29.3%, 33.0% and 36.6%; *p* = 0.049). With regard to family affluence, the proportion of adolescents who needed orthodontic treatment was significantly higher among participants from low affluence families than among those from high affluence families (42.2% in low, 35.1% in medium, and 29.1% in high affluence families; *p* = 0.020). Finally, there was no significant difference in the prevalence of IOTN points between adolescents who were and were not treated for orthodontic anomalies.

Table 3 presents summary statistics of sum scores of the CPQ and its domains. Noticeable values of skewness estimation and differences between the mean and median make the distributions of the sum scores of CPQ and its domains far from normal. From the Variance/Mean ratio we found a great overdispersion for all distributions of sum scores showing them to be even more complicated. The table also compares sum score statistics by gender, age, family affluence, and orthodontic treatment. Regarding gender, females were likely to report higher scores in all domains but significantly so only in the FL and EWB domains as well as in the total CPQ. Sum score statistics of central tendency did not differ significantly by age groups in all domains. Adolescents from high affluence families reported lower EWB and SWB scores compared with adolescents from low affluence families. Comparison of participants’ groups by orthodontic treatment shows that adolescents of the ‘treated’ group were likely to report higher CPQ scores (worse OHRQoL) than their counterparts from the ‘untreated’ group. 

Table 4 displays the results from NBR analysis, which explored the association between the IOTN and CPQ sum scores in bivariate and multivariate (with adjusting predictors) models. Both models revealed that the IOTN has a significant relationship with the overall CPQ sum score and with EWB and SWB sum scores. This association indicates that the increasing demand for orthodontic treatment results in the deterioration of the overall OHRQoL and, particularly, EWB and SWB domains. In the bivariate model, for instance, increasing IOTN by 1 point results in the following average increases: 8.6% (1.086 times) for the CPQ sum score, 15.8% (1.158 times) for the EWB sum score and 20.5% (1.205 times) for the SWB sum score. The associations between the IOTN and sum scores of OS and FL domains were positive but statistically insignificant.

With regard to the socio-demographic predictors, this analysis also revealed several interesting associations, which were in accordance with the results in Table 2. It was seen that females evaluated their OHRQoL as significantly poorer than males in all health domains, except OS. A slight trend in the deterioration of OHRQoL by age was observed, but this remained statistically insignificant in all domains. A greater level of FAS seemed to be associated with lower CPQ sum scores, which suggests that children and adolescents from wealthy families have a higher OHRQoL. This association was significant for overall CPQ and for three of four (except OS) domains. The orthodontic treatment, as it was defined above, had no impact on any of the OHRQoL measures.

The model fit analysis of the hypothesized NBR models, which predicted the CPQ sum scores for the Table 4, suggested that the models were adequate. The goodness-of-fit measures of deviance/df and *χ*^2^/df met the criterion of being close to 1. The omnibus test which compares the fitted model against the intercept-only model revealed that there are significant predictors in the model. The models for domains FL, EWB and SWB showed a generally acceptable fit to the data (using the Poisson regression model, the values of corresponding measures were far from 1). However, both the bivariate and multivariate models for the domain OS fitted poorly, suggesting that the NBR models were not entirely adequate and that any predictor was significantly associated with the sum score of this domain. 

Table 5 shows the magnitude and significance of the relationship between IOTN and CPQ sum scores comparing the gender and age groups. In order to achieve these results, the same NBR model connecting the IOTN and sum scores with adjusted data by family affluence and previous orthodontic treatment was run in groups of males and females combining all ages, and then in three age groups combining both genders. This analysis revealed that the association and, consequently, the effect of malocclusion on OHRQoL is greater among females. For instance, among females, increasing IOTN by 1 point resulted in an average increase of 19.3% (1.193 times) for the EWB sum score mean, while among males this figure was only 7.5% (1.075 times). Although the sample size was balanced between gender groups, among males, the estimations were not significant in either the total CPQ or its domains. 

With regard to the age groups, greater association strength was found in older adolescent groups. For the total CPQ and its OS and FL domains, a statistically significant association was established in the group of 17–18-year-old adolescents only. For the EWB and SWB domains, the association was significant in both younger and older groups. 

## 4. Discussion

The present study is innovative with respect to OHRQoL research in Lithuania. It was a population-based study, using a representative randomized sample of school-aged (11 to 18) adolescents drawn from public schools in Lithuania. Attention was paid to the association between the IOTN (dental health component), which is an objective measure of the severity of malocclusion, and to the CPQ whose sum score is a subjective measure of OHRQoL. The study demonstrated the modest association between these measures. It was revealed that the higher the need for orthodontic treatment, the higher the CPQ scores, and therefore the worse the OHRQoL. Considering the CPQ domains, the strongest relationship was found between IOTN and the emotional and social well-being of adolescents. The study also showed that the strength of the association differed depending on the gender and age of the adolescents.

Due to the high prevalence of malocclusion in young people, growing attention is being paid by researchers interested in examining the impact of this oral disorder on QoL in general and specifically on its OHRQoL component. The literature on this issue has greatly expanded in recent years. For example, a systematic review, published in 2009 by Liu et al. [6], on the association between malocclusion and OHRQoL among children up to 18 years summarized the results of 23 cross-sectional studies, while a Kragt’s et al. [21] recent systematic review and meta-analysis of literature summarized the results of 40 cross-sectional studies. The reviewers claimed evidence for a clear inverse association of malocclusion with OHRQoL. At the same time, they showed that the strength of the association differed depending on the age of children and their cultural environment. Therefore, there is ample opportunity to compare results between this and other work. Since such comparison is hampered by different measurements and methods of data analysis, qualitative comparisons can only be used between studies in which the IOTN and CPQ were used. 

Employing results presented in Table 3 and Table 4 and selecting the Grades of IOTN, the effect of malocclusion can be re-counted into Standardized Mean Difference (SMD) units [21]. For example, a bivariate model for the CPQ in the total sample indicates an increase in the mean sum score of 8.6% (1.086 times) after the increase in IOTN by one point. Comparison of the IOTN Grade 5 with Grade 1, for instance, produces SMD = 0.35 (see: (10.5 × 1.086 × (5 − 1) − 10.5)/10.43 = 0.35). This estimation is greater than (probably due to the selection of adolescents with IOTN marginal grades) but of the same order as SMD = 0.29, which was calculated as a summary measure in meta-analysis to compare children with and without malocclusion in 40 studies [21]. Thus, the results of our study are in agreement with the meta-analysis claiming that malocclusion has a negative impact on the OHRQoL as measured using the CPQ. 

While the general effect of malocclusion on OHRQoL has been established, there is still relatively little evidence for the impact of malocclusion on the sub-domains of OHRQoL. Most often, researchers write about the negative impact of malocclusion on the social and emotional domains of OHRQoL [6,21,52]. The findings of our study support this, agreeing with several previous investigations that revealed a significant relationship between the need for orthodontic treatment and the domains of EWB and SWB [15,53,54]. Our findings also partly confirmed the findings of Spalj et al, who claimed that malocclusion has more impact on EWB than on FL or SWB domains [55]. The possible explanation for this finding is that social life and emotional perceptions play an important role in adolescents’ values. It should be considered that certain occlusal conditions, like visible orthodontic anomalies, can be particularly relevant for adolescents who can become the victims of teasing and bullying [56,57].

In contrast to the EWB and SWB domains, no relationship between malocclusion and the domains of OS and FL was found in the younger adolescence age range (11 to 16 years old), in our study or in the above-mentioned studies [15,53,54,55]. Two possible explanations of this finding have been presented by Simões et al. [15]. According to the authors’ opinion, the first reason is that only very severe occlusal problems would cause impact such domains. In line with this assumption, we tested the impact on the domains comparing increased grades with Grade 1 of the IOTN and found significant associations only for FL and not for OS domain (detailed results are not presented here). Another reason would be that individuals with occlusal problems would probably present oral symptoms and functional limitations in older ages. In our study, this hypothesis was undoubtedly confirmed by finding a strength association between the IOTN and sum scores of the OS and FL domains in the adolescent group of 17–18-years-old (see Table 4). 

In general, we have shown that the age of the adolescents had a major influence on the need for orthodontic treatment and on its association with OHRQoL, even though age had no significant influence on the CPQ scores. Based on our results, it seems that the relationship between malocclusion and OHRQoL changes from being evident in well-being domains only in early adolescence (11–14 years) to becoming evident in all domains in later adolescence (16–18 years). This finding is in contrast with the Masood et al. study which observed a decrease of the impact of malocclusion on OHRQoL as young peoples’ age increased from 15 to 25 years [34]. However, this study recruited individuals older than 18 who may have a real ‘response shift’ with age—the longer individuals live with a malocclusion, the greater the likelihood that they will adjust to its limitations [34]. Longitudinal cohort studies that follow children and adolescents would contribute to a better understanding of the dynamics within the association of malocclusion with OHRQoL [21,58]. To date, no longitudinal study has been conducted to follow subjects from 12 to 18-years-old [13]. 

We also examined gender differences. Literature provides evidence that the subjective demand for orthodontic treatment is higher among females than males in adolescence [59]. In contrast, we did not see a gender difference in the severity of malocclusion objectively assessed using the IOTN-DHC. Several studies have also demonstrated that compared with males, females had more negative experiences in emotional and social wellbeing domains [13,14]. Results of our study supported these findings confirming the gender difference in OHRQoL. In part, this may be explained by the fact that the impact of malocclusion on the OHRQoL was much greater among the females. Even after gender differences were demonstrated in the association between malocclusion and OHRQoL and were found to be significant across three domains (FL, EWB and SWB), we still do not possess a full understanding of why this is so. There is also a lack of literature both confirming and/or explaining this fact.

In this paper we explored modelling data with techniques that estimate the ratio of the sum score means. Such an approach provides correct estimates and is a better alternative for the analysis of cross-sectional studies than logistic regression modelling, since the estimate is more interpretable than the odds ratio [60]. Another advantage of our study is that we have controlled the model to fit to the existing data. It was shown that the models which were applied in the present data analysis were robust and have not been affected by high levels of skewness and overdispersion in outcome variables (sum scores of CPQ and its domains). The quality of models was controlled with the deviance value. However, goodness-of-fit characteristics of the OS models, both of the univariate and multivariate model, were unacceptable (deviance/df differed from 1 too much). An incorrectly specified model may be one of the reasons why the search for an association was unsuccessful [50,51].

There are several limitations to our study that should be mentioned, in particular the assessment of OHRQoL which was based on a self-report that might be affected by recall bias as well as social norms. To overcome this bias, the participants were assured anonymity and confidentiality, and the questions were pre-tested before conducting the actual survey. We used the original CPQ questionnaire with 37 questions, the version which was validated among adolescents aged 11–14 years in many languages. The validity of the Lithuanian version of the CPQ was tested among adolescents up to the ages of 18 [45]. Kragt‘s et al. review [21] found that this instrument has been used in 20 of 40 studies to examine the association between malocclusion and OHRQoL, although little is known about psychometric characteristics of this instrument for older adolescents. We operated under the assumption that the CPQ is valid for older adolescents at least as much as for those aged 11–14 years. Partially, this assumption was supported by findings of the present study which showed increased strength of the relationship between IOTN and CPQ sum scores for older adolescents. Some facts on this issue were also revealed in our previous publication which demonstrated a significant correlation between CPQ scores and global life satisfaction in all age groups of adolescents [46]. 

Another limitation concerns the measurements of severity of malocclusion as only the IOTN-DHC was chosen for this purpose. To date, there is no evidence-based method of quantification for malocclusion in children and adolescents as they lack assessment of all occlusal traits [24,25]. Consequently, the studies of association between OHRQoL and malocclusion in young people were based on different occlusal indices providing controversial findings, either the strength of the association was weak, or due to methodological issues, findings were not conclusive [13,31,32,39]. Despite all these contradictions, subjects in most studies were classified according to their orthodontic treatment need rather than by occlusial traits [6,21,39]. Although the IOTN-DHC is valid and reliable instrument, it may be a relatively insensitive instrument to measure minor occlusial traits and irregularities which mostly affect patients’ appearance and about which a patient is deeply concerned [61,62]. Another limitation of this instrument is that only the highest scoring trait is used to assess treatment need. Other measures have also been frequently chosen [6,21]. One of these measures, ICON [63], has been also recorded during dental examination in our study. Unfortunately, we were not able to focus on more indicators of malocclusion severity or on more personal factors which might influence the relationship between malocclusion and OHRQoL due to the limited scope of the paper. The analysis of these data will be continued at a later date. 

The choice of the IOTN-DHC to measure adolescent malocclusion was also related to the mixed dentition which occurred among adolescents aged 11–14 years. It has been noticed that the IOTN in the mixed dentition stage can present a tendency to overestimate the malocclusion [64]. However, in our study the cumulative rate of IOTN-DHC Grades 4 and 5 in this age group was the lowest (29.3%) of all three age groups of adolescents. The fact that IOTN-DHC is based on the most severe malocclusion trait and that in this age the overjet was most often observed trait, which is not related to mixed dentition, could have helped to avoid the risk of overestimation. Therefore, the figures of IOTN-DHC among adolescents aged 11–14 years are comparable with corresponding figures of older adolescents.

The study may present a selection bias due to differences between the characteristics of the adolescents treated for orthodontic anomalies and those of untreated, irrespectives of employed data adjusting for orthodontic treatment. The revealed differences in the CPQ sum scores between these groups with worse OHRQoL among adolescents from the ‘treated’ group require further analysis. Some aspects of this issue have been already discussed in our previous publication [46] and will be continued at a later date. The reviews of the impact of malocclusion and its treatment on OHRQoL provide evidence that patients who need orthodontic treatment are concerned with improving their appearance and social acceptance, often more than they are with improving their oral health and function [64].

Finally, the perceived limitation might be lower than the calculated sample size for 11–14-year-olds. However, the available number of participants proved to be adequate, having appropriate sample power to identify the impact of orthodontic treatment need in EWB and SWB domains.

Despite these limitations, we believe that the current findings provide further evidence on the relationship between malocclusion and OHRQoL and help better the understanding regarding the impact of oral health conditions on the life of an adolescent. This can help in better treatment planning and better allocation of resources, as oral health perceptions can vary for adolescents by gender, age and, likely, other factors.

## 5. Conclusions

This is the first study to measure OHRQoL in Lithuanian adolescents and to examine its relationship with malocclusion. The relationship was examined in a representative sample of adolescents aged 11 to 18 years. The study demonstrated the modest association between IOTN and OHRQoL, suggesting a negative impact of malocclusion on the OHRQoL. The higher impact occurred in the emotional and social well-being domains. Females and older adolescents suffered from malocclusion more severely than males and younger counterparts. 

## Figures and Tables

**Table 1:** Distribution of the study participants by socio-demographic variables and orthodontic treatment (*N* = 911).

	Study Sample	
	*N*	(%)
*Gender:*		
Boys	370	(40.6)
Girls	541	(59.4)
*Age:*		
11–14-year-old	195	(21.4)
15–16-year-old	418	(45.9)
17–18-year-old	298	(32.7)
*Family affluence:*		
Low	109	(12.3)
Medium	353	(39.7)
High	426	(48.0)
Missing	23	
*Dental plate:*		
Present	45	(15.0)
Previous	190	(21.0)
Never	676	(74.0)
*Fixed orthodontic appliance:*		
Present	26	(2.9)
Previous	33	(3.6)
Never	852	(93.5)
*Present or previous orthodontic treatment with dental plate or fixed orthodontic appliance:*		
Untreated	644	(70.7)
Treated	267	(29.3)

**Table 2:** Distribution of the study participants by the Index of Orthodontic Treatment Need (IOTN) grades for the total sample (*N* = 911) and for groups by gender, age, family affluence, and previous orthodontic treatment.

	Grades										*p* ^2^
	1		2		3		4 ^1^		5 ^1^		
	*N*	(%)	*N*	(%)	*N*	(%)	*N*	(%)	*N*	(%)	
Total sample	109	(12.0)	275	(30.2)	223	(24.5)	255	(28.0)	49	(5.4)	
*Gender:*											0.557
Boys	43	(11.6)	104	(28.1)	96	(25.9)	110	(29.7)	17	(4.6)	
Girls	66	(12.2)	171	(31.6)	127	(23.5)	145	(26.8)	32	(5.9)	
*Age:*											**0.038**
11–4-year-old	20	(10.3)	63	(32.3)	55	(28.2)	45	(23.1)	12	(6.2)	
15–16-year-old	45	(10.8)	121	(28.9)	114	(27.3)	113	(27.0)	25	(6.0)	
17–18-year-old	44	(14.8)	91	(30.5)	54	(18.1)	97	(32.6)	12	(4.0)	
*Family affluence:*											0.182
Low	12	(11.0)	28	(25.7)	23	(21.1)	37	(33.9)	9	(8.3)	
Medium	33	(9.3)	109	(30.9)	87	(24.6)	105	(29.7)	19	(5.4)	
High	60	(14.1)	133	(31.2)	109	(25.6)	106	(24.9)	18	(4.2)	
*Orthodontic treatment:*											0.145
Untreated	78	(12.1)	204	(31.7)	162	(25.2)	171	(26.6)	29	(4.5)	
Treated	31	(11.6)	71	(26.6)	61	(22.8)	84	(31.5)	20	(7.5)	

^1^ Regarded as the need for treatment; ^2^ χ^2^ test; *p* ≤ 0.05 are in bold.

**Table 3:** Summary statistics of sum scores of the CPQ and its domains for the total sample (*N* = 911) and for groups by gender, age and family affluence.

CPQ/Domain ^1^	Group	Mean	SD	Median	IQR	*p* ^2^
CPQ Variance = 108.79 Skewness = 1.95	Total sample	10.50	10.43	7	11	
	*Gender:*					
	Boys	9.00	9.15	6	9	0.001
	Girls	11.53	11.11	8	11	
	*Age:*					
	11–14-year-old	9.93	10.25	7	10	0.256
	15–16-year old	10.54	10.31	7	12	
	17–18-year-old	10.82	10.74	8	10	
	*Family affluence:*					
	Low	13.48	11.85	10	12	0.201
	Medium	10.66	10.67	7	10	
	High	9.79	9.84	7	11	
	*Orthodontic treatment:*				
	Untreated	9.86	9.75	7	10	0.041
	Treated	12.06	11.78	8	12	
OS Variance = 8.72 Skewness = 0.977	Total sample	4.01	2.95	3	4	
	*Gender:*					
	Boys	3.79	2.85	3	3	0.534
	Girls	4.16	3.02	3	4	
	*Age:*					
	11–14-year-old	3.74	2.79	3	3	0.588
	15–16-year old	3.98	2.96	3	4	
	17–18-year-old	4.23	3.03	4	4	
	*Family affluence:*					
	Low	4.44	3.19	4	4	0.189
	Medium	4.07	2.76	4	4	
	High	3.86	2.97	3	4	
	*Orthodontic treatment:*				
	Untreated	3.84	2.83	3	3	0.363
	Treated	4.42	3.20	4	4	
FL Variance = 8.71 Skewness = 2.48	Total sample	1.94	2.95	1	3	
	*Gender:*					
	Boys	1.71	2.75	0	2	0.042
	Girls	2.10	3.08	1	3	
	*Age:*					
	11–14-year-old	1.76	2.76	1	2	0.531
	15–16-year old	2.08	3.07	1	3	
	17–18-year-old	1.86	2.91	1	3	
	*Family affluence:*					
	Low	2.68	3.19	4	4	0.086
	Medium	1.96	2.80	1	3	
	High	1.81	2.84	1	2	
	*Orthodontic treatment:*				
	Untreated	1.77	2.72	1	2	0.160
	Treated	2.35	3.41	1	3	
EWB Variance = 24.00 Skewness = 2.44	Total sample	3.30	4.90	2	4	
	*Gender:*					
	Boys	2.29	3.71	0	3	<0.001
	Girls	3.99	5.47	2	5	
	*Age:*					
	11–14-year-old	3.30	5.12	2	4	0.998
	15–16-year old	3.14	4.70	1	5	
	17–18-year-old	3.51	5.04	2	4	
	*Family affluence:*					
	Low	4.39	4.64	3	6	<0.001
	Medium	3.34	5.06	2	4	
	High	3.05	4.88	1	4	
	*Orthodontic treatment:*				
	Untreated	3.07	4.63	1	4	0.186
	Treated	3.85	5.46	2	5	
SWB Variance = 8.57 Skewness = 3.81	Total sample	1.25	2.93	0	1	
	*Gender:*					
	Boys	1.20	2.91	0	1	0.170
	Girls	1.29	2.94	0	1	
	*Age:*					
	11–14-year-old	1.13	2.80	0	1	0.548
	15–16-year old	1.33	2.98	0	2	
	17–18-year-old	1.22	2.94	0	1	
	*Family affluence:*					
	Low	1.94	3.83	0	2	0.049
	Medium	1.29	3.25	0	1	
	High	1.08	2.37	0	1	
	*Orthodontic treatment:*				
	Untreated	1.17	2.84	0	1	0.155
	Treated	1.44	3.13	0	2	

^1^ Here, variance and skewness are presented for the total sample. ^2^ Median test. SD: standard deviation; IQR: interquartile range; CPQ: Child Perception Questionnaire; OS: Oral Symptoms; FL: Functional Limitations; EWB: Emotional Wellbeing; SWB: Social Wellbeing. *p* ≤ 0.05 are in bold.

**Table 4:** Relationship between CPQ/Domain sum scores and Index of Orthodontic Treatment Need, using Negative Binomial Regression models.

CPQ/Domain	Model	Compared Groups ^1^	*p*	RSSM	95% CI for RSSM	
					Lower	Upper
CPQ	Bivariate	IOTN	**0.006**	1.086	1.024	1.152
	Multivariate	IOTN	**0.016**	1.074	1.013	1.138
		Girls vs. Boys	**0.002**	1.238	1.085	1.413
		Age3 vs. Age1	0.523	1.062	0.883	1.278
		Age2 vs. Age1	0.533	1.058	0.896	1.262
		FAS3 vs. FAS1	**0.002**	0.743	0.613	0.905
		FAS2 vs. FAS1	**0.015**	0.785	0.645	0.955
		Treated vs. Untreated	0.122	1.131	0.968	1.321
OS	Bivariate	IOTN	0.398	1.019	0.975	1.066
	Multivariate	IOTN	0.774	1.013	0.969	1.058
		Girls vs. Boys	0.186	1.069	0.968	1.180
		Age3 vs. Age1	0.052	1.138	0.999	1.297
		Age2 vs. Age1	0.282	1.071	0.945	1.213
		FAS3 vs. FAS1	0.079	0.871	0.747	1.016
		FAS2 vs. FAS1	0.218	0.909	0.781	1.058
		Treated vs. Untreated	0.083	1.104	0.987	1.235
FL	Bivariate	IOTN	0.441	1.038	0.944	1.141
	Multivariate	IOTN	0.512	1.031	0.941	1.129
		Girls vs. Boys	0.077	1.201	0.980	1.472
		Age3 vs. Age1	0.935	1.012	0.762	1.343
		Age2 vs. Age1	0.267	1.163	0.891	1.518
		FAS3 vs. FAS1	**0.012**	0.675	0.497	0.918
		FAS2 vs. FAS1	**0.038**	0.720	0.528	0.982
		Treated vs. Untreated	0.206	1.170	0.918	1.491
EWB	Bivariate	IOTN	0.001	1,158	1.062	1.262
	Multivariate	IOTN	**0.003**	1.140	1.047	1.241
		Girls vs. Boys	**<0.001**	1.671	1.363	2.049
		Age3 vs. Age1	0.936	1.012	0.759	1.349
		Age2 vs. Age1	0.624	0.934	0.712	1.226
		FAS3 vs. FAS1	**0.011**	0.722	0.561	0.929
		FAS2 vs. FAS1	**0.023**	0.745	0.578	0.960
		Treated vs. Untreated	0.177	1.172	0.931	1.476
SWB	Bivariate	IOTN	**0.005**	1.205	1.058	1.373
	Multivariate	IOTN	**0.008**	1.189	1.045	1.351
		Girls vs. Boys	0.880	1.024	0.755	1.389
		Age3 vs. Age1	0.812	1.054	0.684	1.625
		Age2 vs. Age1	0.460	1.164	0.778	1.743
		FAS3 vs. FAS1	**0.015**	0.580	0.373	0.900
		FAS2 vs. FAS1	0.067	0.652	0.412	1.030
		Treated vs. Untreated	0.612	1.095	0.771	1.556

^1^ Age1: 11–14-year-old; Age2: 15–16-year-old; Age3: 17–18-year-old; FAS1: low level of family affluence; FAS2: medium level of family affluence; FAS3: high level of family affluence. IOTN: Index of Orthodontic Treatment Need; CPQ: Child Perception Questionnaire; OS: Oral Symptoms; FL: Functional Limitations; EWB: Emotional Wellbeing; SWB: Social Wellbeing; RSSM: Ratio of Sum Score Means; CI: Confidence Interval. *p* ≤ 0.05 are in bold.

**Table 5:** Relationship between CPQ/Domain sum scores and Index of Orthodontic Treatment Need in groups of adolescents by gender and age ^1^.

CPQ/Domain	Compared Groups	*p*	RSSM	95% CI for RSSM	
				Lower	Upper
CPQ	*Gender:*				
	Boys	0.904	1.003	0.949	1.069
	Girls	<0.001	1.122	1.066	1.18
	*Age:*				
	11–14-year-old	0.226	1.045	0.976	1.118
	15–16-year-old	0.867	1.005	0.946	1.067
	17–18-year-old	<0.001	1.199	1.134	1.268
OS	*Gender:*				
	Boys	0.066	0.97	0.923	1.019
	Girls	0.026	1.047	1.005	1.089
	*Age:*				
	11–14-year-old	0.675	0.99	0.944	1.038
	15–16-year-old	0.333	0.977	0.932	1.024
	17–18-year-old	0.001	1.075	1.031	1.122
FL	*Gender:*				
	Boys	0.492	0.97	0.889	1.058
	Girls	0.093	1.072	0.988	1.163
	*Age:*				
	11–14-year-old	0.09	1.097	0.986	1.221
	15–16-year-old	0.117	0.928	0.845	1.019
	17–18-year-old	<0.001	1.168	1.074	1.27
EWB	*Gender:*				
	Boys	0.102	1.075	0.986	1.173
	Girls	<0.001	1.193	1.111	1.281
	*Age:*				
	11–14-year-old	0.049	1.089	1.001	1.185
	15–16-year-old	0.181	1.062	0.972	1.161
	17–18-year-old	<0.001	1.315	1.206	1.434
B	*Gender:*				
	Boys	0.212	1.076	0.959	1.208
	Girls	<0.001	1.259	1.121	1.414
	*Age:*				
	11–14-year-old	0.049	1.099	1.001	1.208
	15–16-year-old	0.037	1.09	1.005	1.182
	17–18-year-old	<0.001	1.412	1.3	1.534

^1^ Data adjusted by family affluence and orthodontic treatment, and weighted by sample size. CPQ: Child Perception Questionnaire; OS: Oral Symptoms; FL: Functional Limitations; EWB: Emotional Wellbeing; SWB: Social Wellbeing; RSSM: ratio of sum score means; CI: Confidence Interval. *p* ≤ 0.05 are in bold.

## Abbreviations

The following abbreviations are used in this manuscript:

AC	Aesthetic Component
CI	Confidence Interval
CPQ	Child Perceptions Questionnaire
DHC	Dental Health Component
EWB	Emotional Well-being
FAS	Family Affluence Scale
FL	Functional Limitations
ICON	Index of Complexity Outcome and Need
IOTN	Index of Orthodontic Treatment Need
IQR	Interquartile Range
NBR	Negative Binomial Regression
OHRQoL	Oral Health-Related Quality of Life
OS	Oral Symptoms
RSSM	Ratio of Sum Score Means
QoL	Quality of Life
SD	Standard Deviation
SMD	Standardized Mean Difference
SWB	Social Well-being
WHO	World Health Organization
